# Thiophene structure influences plasmonic creatinine sensing through molecular interaction and surface morphology

**DOI:** 10.1038/s41598-025-27141-3

**Published:** 2025-12-02

**Authors:** Nurul Izzah Zakaria, Nur Afifah Ahmad Nazri, Nur Hidayah Azeman, Muhammad Asif Ahmad Khushaini, Muhammad Qayyum Othman, Mohd Hafiz Abu Bakar, Tengku Hasnan Tengku Abdul Aziz, Mohd Suzeren Md Jamil, Ahmad Rifqi Md Zain, Ahmad Ashrif A Bakar

**Affiliations:** 1 Institute of Microengineering and Nanoelectronics (IMEN), Universiti Kebangsaan Malaysia, Bangi, 43600 Selangor Malaysia; 2 Faculty of Science and Technology, Universiti Kebangsaan Malaysia, Bangi, 43600 Selangor Malaysia; 3 Photonics Technology Laboratory, Department of Electrical, Electronic, and Systems Engineering, Faculty of Engineering and Built Environment, Universiti Kebangsaan Malaysia, 43600 Bangi, Selangor Malaysia; 4 Institute of Power Engineering, Universiti Tenaga Nasional, Jalan IKRAM- UNITEN, Putrajaya Campus, Kajang, 43000 Selangor Malaysia

**Keywords:** Chemistry, Materials science, Nanoscience and technology, Optics and photonics

## Abstract

This study investigates the influence of molecular structure on plasmonic sensing performance by comparing two thiophene-based small molecules, benzo[b]thiophene-2-carboxaldehyde (BTCA) and tetrahydrothiophene (THT), as sensing interfaces for label-free creatinine detection. Surface plasmon resonance (SPR) measurements, supported by Fourier-transform infrared spectroscopy (FTIR), field emission scanning electron microscopy (FESEM), energy-dispersive X-ray spectroscopy (EDX), and atomic force microscopy (AFM), reveal distinct differences in analyte interaction and interfacial behavior. BTCA, featuring a conjugated aromatic aldehyde structure, enables stronger hydrogen bonding and dipole–dipole interactions with creatinine, evidenced by spectral changes, vibrational mode suppression, and uniform surface morphology. These interactions contribute to a more linear and consistent SPR response (R² = 0.97) compared to THT, which exhibits weaker molecular interactions, disordered surface features, and a segmented sensing profile. This comparative analysis highlights how the molecular structure of thiophene derivatives influences interfacial interaction and sensing behavior. The findings provide experimental evidence that structural differences between BTCA and THT result in distinct surface morphologies and optical responses, offering practical guidance for selecting suitable sensing layers in plasmonic creatinine detection.

## Introduction

Chronic kidney disease (CKD) poses a significant global health burden, particularly among aging populations and individuals with diabetes or hypertension^1^. Accurate and timely detection of renal dysfunction is essential for effective clinical intervention. Creatinine, a byproduct of muscle metabolism, is a widely accepted biomarker for evaluating kidney function. However, conventional diagnostic methods rely on serum or urine creatinine analysis, which requires invasive blood collection or cumbersome sample handling, thereby limiting their suitability for rapid or patient-friendly screening^2^. Saliva has emerged as a potential alternative matrix for non-invasive creatinine monitoring^3^. Despite its clinical appeal, current analytical methods often lack the sensitivity required to detect low creatinine levels in the early stages of CKD. This limitation underscores the need for novel sensing platforms that can achieve high sensitivity and specificity at physiologically relevant concentrations^4,5^.

Surface plasmon resonance (SPR) has gained traction as a powerful platform for biomolecular sensing due to its label-free, real-time detection capabilities and high sensitivity to small refractive index changes at metal-dielectric interfaces^6,7^. This technique is particularly suited for detecting trace levels of analytes in complex biological fluids, such as saliva, offering a non-invasive alternative to traditional diagnostic approaches^8^. Ongoing advances in surface functionalization have expanded the applicability of SPR to small-molecule detection by minimizing the need for labeling or elaborate sample preparation. In an SPR sensor, molecular interactions at the interface modulate the local refractive index, resulting in a measurable shift in the SPR resonance wavelength that correlates with analyte concentration^9^. Despite its advantages, SPR remains inherently less responsive to low-molecular-weight compounds such as creatinine, due to their limited mass and weak optical contrast. Overcoming this limitation requires the development of engineered sensing surfaces that enhance molecular recognition and efficiently transduce subtle interfacial changes into optical signals^10^.

Thiophene-based molecules are structurally ideal for plasmonic sensing due to their electron-rich π-conjugated systems and strong binding affinity to noble metal surfaces such as gold. Their ability to form chemically stable, uniform films and engage in non-covalent molecular recognition makes them highly suitable for detecting small molecules. Despite these advantages, their application in surface plasmon resonance sensing remains largely unexplored. Covalent routes such as aryl diazonium electro grafting and N-heterocyclic carbenes (NHCs) yield highly stable Au interfaces for biosensing, including SPR^11,12^, but thiophenes interact through weaker Au–S and π–π interactions; yet, their π-conjugation and sulfur-rich structures provide enhanced polarizability and molecular recognition sites, which are valuable for plasmonic sensing. In this study, we introduce thiophene derivatives, benzo[b]thiophene-2-carboxaldehyde (BTCA) and tetrahydrothiophene (THT), as a new class of small-molecule organic coatings for SPR-based detection of creatinine. BTCA incorporates a polar aldehyde group that facilitates directional hydrogen bonding with creatinine’s amine groups, while THT contributes through dipole–dipole and electrostatic interactions^13^. These complementary mechanisms provide molecular specificity that is absent in bare gold and difficult to achieve with conventional modifiers such as carbon nanomaterials or polymer films, which often suffer from swelling, degradation, or limited selectivity^14^. The sulfur-rich and π-conjugated structure of thiophene enables strong surface adhesion to gold and enhanced polarizability, which collectively facilitate efficient coupling with the surface plasmon field during analyte recognition.

Using a Kretschmann-configuration SPR system, we demonstrate label-free creatinine detection across physiologically relevant concentrations (0–1.2 mg/dL). Supporting characterizations using FTIR, AFM, FESEM, and EDX confirm the chemical interaction and morphological transformation of the thiophene films upon binding with creatinine. To the best of our knowledge, this is the first comprehensive investigation of thiophene-based sensing layers in surface plasmon resonance biosensing. The distinct molecular structures of BTCA and THT, differing in aromaticity, functional groups, and polarity, enable a direct comparison of how chemical architecture influences molecular recognition, interfacial behavior, and signal transduction. These findings establish thiophene derivatives as a chemically tunable, stable, and scalable platform for non-invasive renal diagnostics, while providing a structure-guided framework for designing plasmonic interfaces tailored to specific biomolecular interactions.

## Methods

### Materials

All reagents used in this study were purchased from commercial sources (Merck Millipore). Benzo(b)thiophene-2-carboxaldehyde (≥ 97%) and tetrahydrothiophene (≥ 98%) were used as the primary material to develop the optical sensing substrate, while creatinine anhydrous (≥ 98%) was used as the analyte.

## Preparation of gold sputter coating

Gold sputtering was performed on a 24 mm x 24 mm microscope slide. The slide was first cleaned with deionized water and dried with ethanol. Next, a 50 nm Au layer was deposited onto the slide using a sputtering machine with a current of 60 mA and a sputtering rate of 9 nm/min for 60 s, utilizing a tooling factor of 2.30. The sputter coating process was repeated five times to achieve a robust sample with a gold layer thickness of 40–50 nm.

## Preparation of sensing materials coating

A gold-coated microscope slide (substrate) contains two sensing materials: benzo(b)thiophene-2-carboxaldehyde and tetrahydrothiophene. 600 µL of these thiophene derivatives was drop-cast onto a gold-coated microscope slide and dried in a laboratory oven for 10–20 min at 137–235 °C to form the optical sensing substrate.

## Characterization of sensing materials coating

Fourier-transform infrared (FTIR) spectra were obtained using a Perkin-Elmer Spectrum 2000 ATR-FTIR spectrometer (Perkin-Elmer, USA) with a scanning range of 650–4000 cm⁻¹ and a resolution of 2 cm⁻¹. Atomic force microscopy (AFM) images were acquired using a Bruker Dimension Icon (Bruker, USA) in tapping mode over scan areas of 1–5 μm² and analyzed with NanoScope software. Field emission scanning electron microscopy (FESEM) was carried out using a TESCAN CLARA GM microscope (TESCAN, Czech Republic) with a magnification capability of 2–5 × 10⁶ and resolution down to the nanometer scale. Energy-dispersive X-ray spectroscopy (EDX) was performed using an Oxford Instruments detector coupled to the FESEM, under high-vacuum conditions at an accelerating voltage of 5 kV. Elemental mapping was carried out with a step size of 1 μm.

## Testing the optical sensing substrate using the SPR sensor

**Fig. 1:**
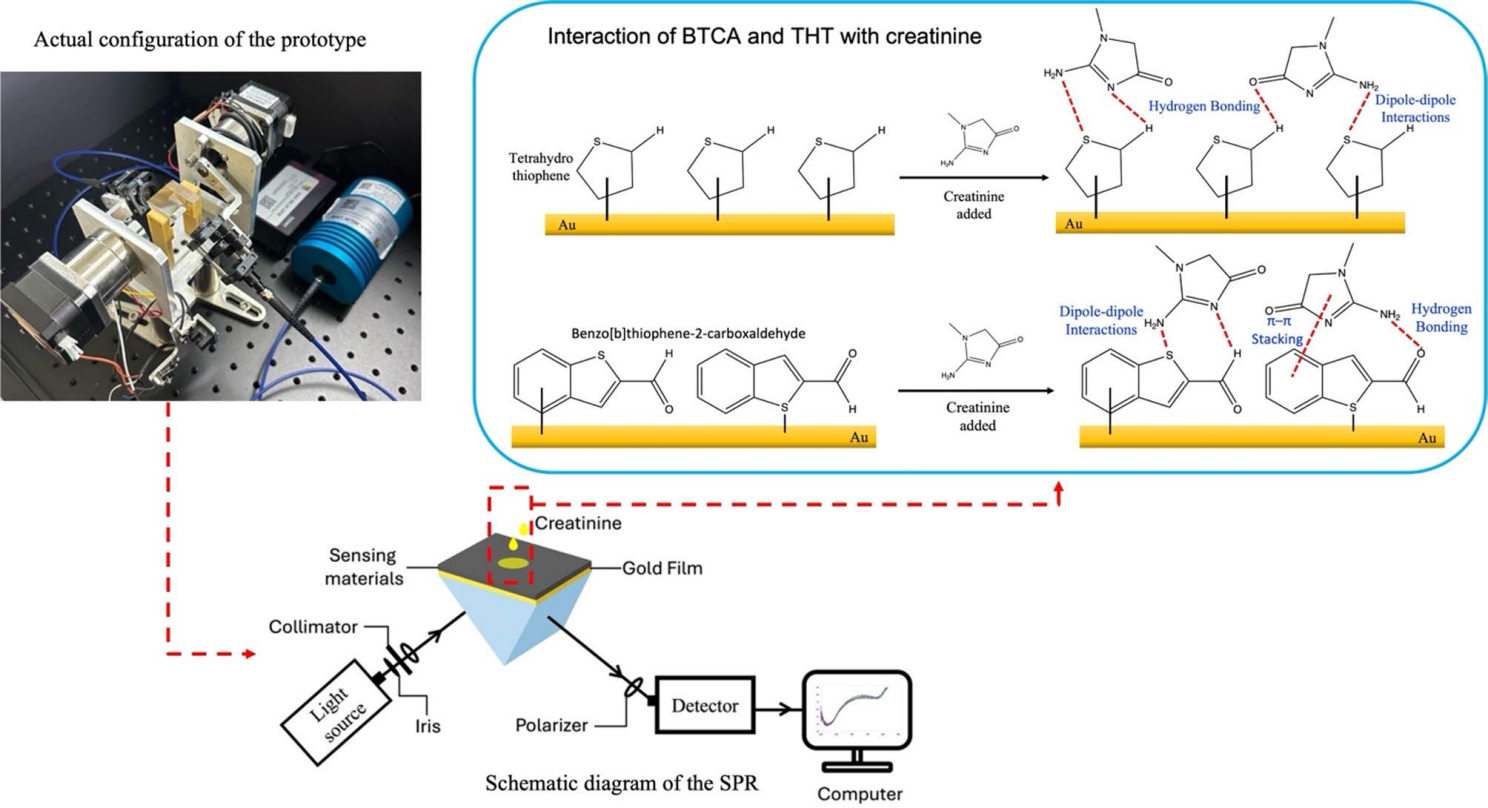
Actual configuration and schematic diagram of the prototype Kretschmann-based SPR sensing system employing BTCA and THT as thiophene-derived sensing layers for creatinine detection.

Surface plasmon resonance (SPR) measurements were performed using a prototype setup configured in the Kretschmann geometry, as depicted in Fig. 1. Illumination was provided by a Halogen Light Source (HL-2 2000, Ocean Optics, USA), delivered through a 400 μm core diameter multimode fiber optic patch cord (SMA-SMA, Thorlabs, USA). The light beam was passed through a polarizer (Thorlabs, USA) with a wavelength range of 400–1100 nm to obtain p-polarized light, which is essential for SPR excitation. An iris was used to control the beam spot size. The p-polarized light was directed at a fixed incident angle of 53.8°, which was theoretically optimized for efficient SPR excitation, onto an SF11 right-angle prism. A 50 nm gold (Au) thin film was deposited on a glass substrate, which was mounted with the glass side in contact with the prism and the Au surface exposed for analyte interaction. A 25 µL droplet of creatinine solution was applied onto the sensing surface. Reflected light from the interface was collected by a spectrometer (YIXIST, Shanghai Yixist Technology Co., China), and spectral data were analyzed using the YSV software (Yixist, China). The measurements were conducted across creatinine concentrations ranging from 0.2 to 1.2 mg/dL—all measurements at ~ 25 °C; creatinine solutions were prepared in DI water (nominal near-neutral pH). Comparative analyses were carried out using Au films functionalized with THT and BTCA. The resulting wavelength shifts in the reflectance spectra were used to construct calibration plots. All SPR measurements were performed in triplicate to assess precision, and error bars represent the standard deviation across independent trials. The limit of detection (LOD) and limit of quantification (LOQ) were calculated based on the standard deviation of the response (σ) and the slope (S) of the calibration curve using the formulas LOD = 3σ/S and LOQ = 10σ/S. These metrics reflect the system’s measurement uncertainty and demonstrate its suitability for detecting low concentrations of creatinine. The linear regression analysis (R² > 0.96) further confirms the accuracy and consistency of the sensor’s response.

## Results

### Molecular study

**Fig. 2:**
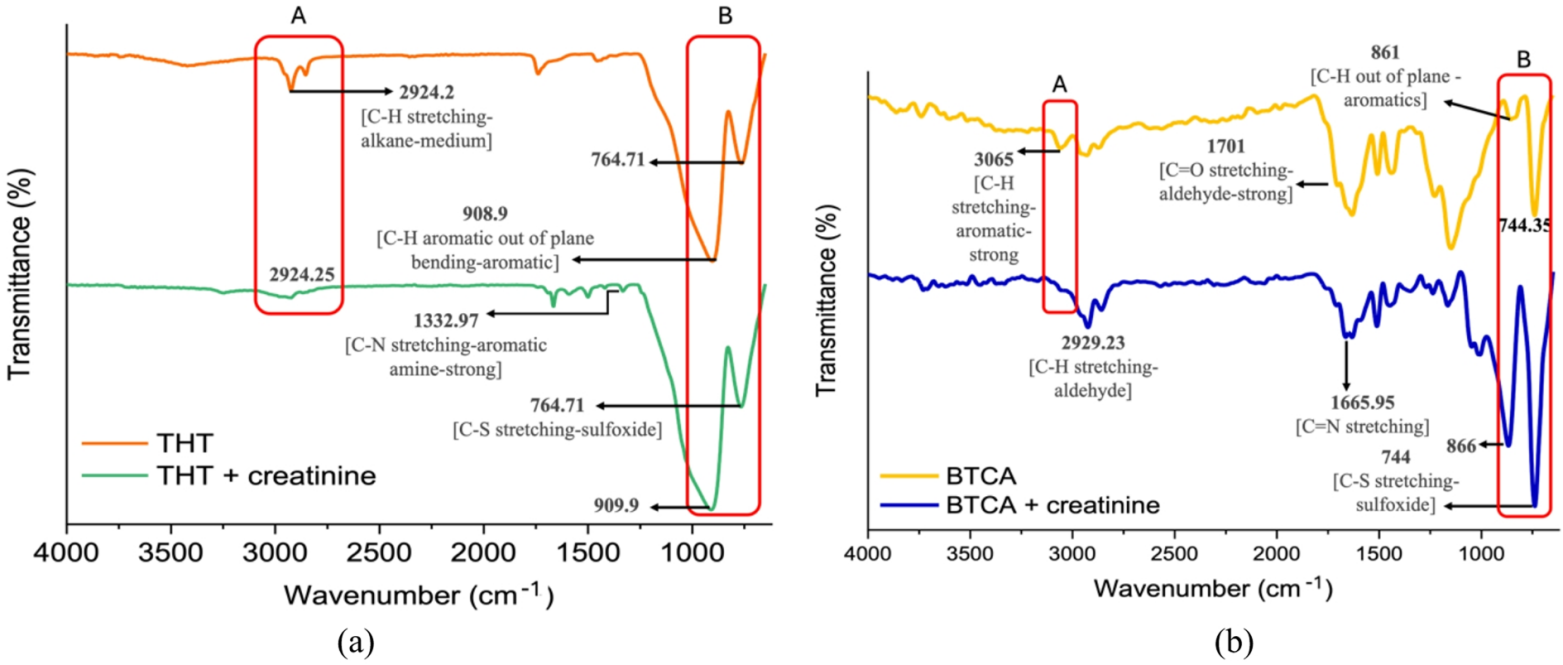
FTIR spectra of (**a**) THT and (**b**) BTCA before and after the addition of creatinine; red boxes highlight regions with notable post-exposure spectral changes.

The gold FTIR spectra presented in Fig. 2 provide evidence of molecular interactions between tetrahydrothiophene, benzo[b]thiophene-2-carboxaldehyde, and creatinine, as indicated by characteristic vibrational shifts. As shown in Fig. 2a, the spectrum of pure THT (orange line) exhibits characteristic peaks at 2924 cm⁻¹ corresponding to C–H stretching of alkanes, and at 764 cm⁻¹ attributed to C–S stretching of sulfoxide, confirming the presence of the thiophene ring. Upon the addition of creatinine (green line), noticeable spectral changes are observed. Notably, a new peak appears at 1332 cm⁻¹, corresponding to strong C–N stretching of an aromatic amine, clearly indicating the incorporation of creatinine. Additionally, the peak from 908 to 909 cm⁻¹ associated with C–H out-of-plane bending in aromatic systems becomes more pronounced, and the intensity of the sulfoxide (C–S) band also slightly changes. In BTCA, Fig. 2b exhibits a similar phenomenon, where the intensity of the 744 cm⁻¹ C-S stretching of the sulfoxide increases (blue line), but the peak remains unchanged^15^. The same applies to the peak of C-H stretching at 2929 cm⁻¹. One distinct peak of C–H out-of-plane in aromatic (yellow line) becomes a more prominent peak and shifts from 861 cm⁻¹ to 866 cm⁻¹. Unlike THT, BTCA exhibited distinct peaks corresponding to C = N, C = O, and C–H stretching at 1665 cm⁻¹, 1701 cm⁻¹, and 3065 cm⁻¹, respectively, which showed reduced intensity after interaction. In the spectrum of pure BTCA, the absorption band at 1701 cm⁻¹ corresponds to the C = O stretching of the aldehyde group, which may form via oxidation of the thiophene moiety upon air exposure^16^. These spectral modulations indicate more pronounced perturbations for BTCA than for THT, consistent with the presence of a conjugated aromatic aldehyde group. Overall, the vibrational shifts and intensity changes are compatible with the involvement of polar functional groups and aromatic domains at the interface. While not definitive proof of a specific binding mode, the data indicate structure-dependent interactions driven by hydrogen bonding and dipolar effects, aligning with the enhanced sensing performance observed in SPR for BTCA^6,17^.

## Surface morphology study

**Fig. 3:**
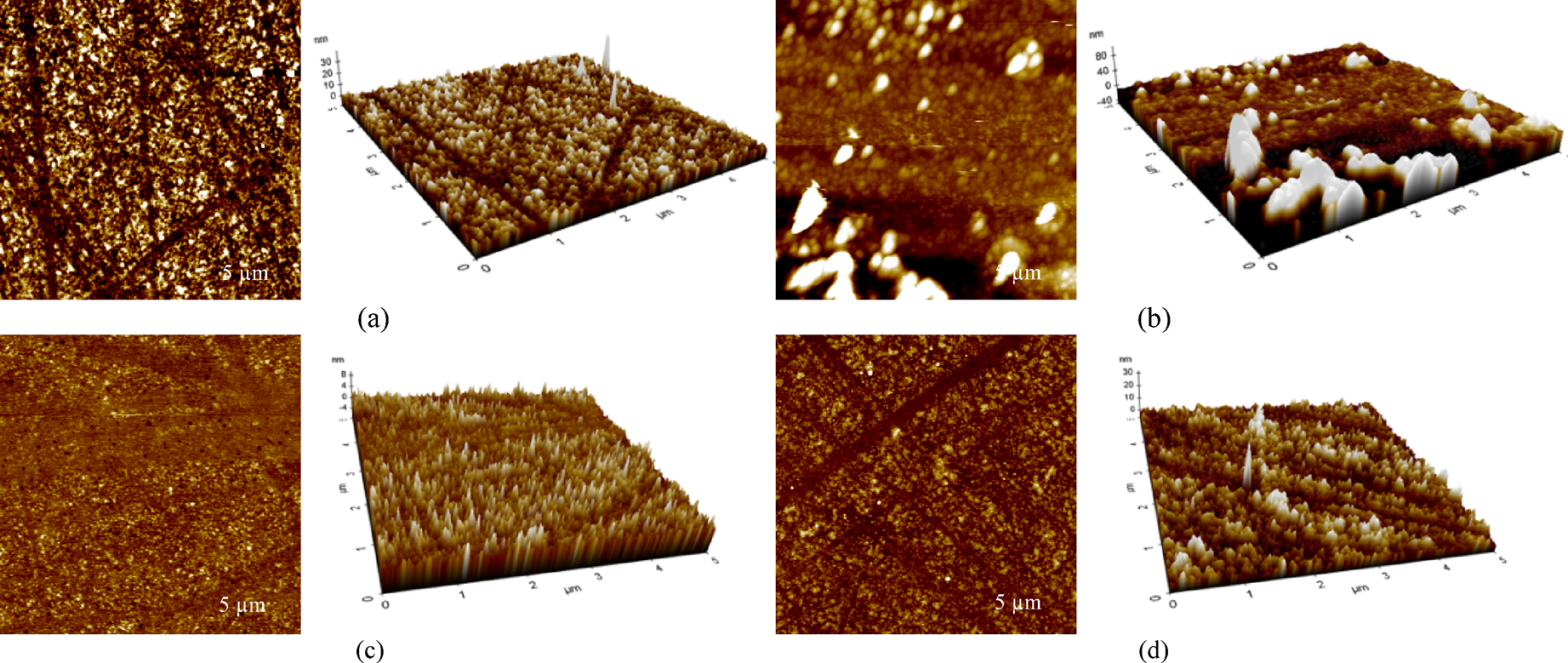
AFM topography images of (**a**) THT-before, (**b**) THT-after, (**c**) BTCA-before and (**d**) BTCA-after creatinine exposure. Acquisition: 5 × 5 μm, 256 pxl, 1 Hz. Each panel includes a 3D inset (same field of view and Z scale (nm)).

The AFM analysis provides detailed insight into the surface morphology of THT and BTCA-coated gold films before and after the introduction of creatinine, with the inset of 3D images. The surface of tetrahydrothiophene (Fig. 2a) alone represents a relatively smooth texture with a low root mean square roughness (Rq: 2.157 nm). Upon exposure to creatinine, as shown in Fig. 3b, the surface roughness (Rq: 10.198 nm) of THT increases significantly after introducing creatinine. Meanwhile, a noticeable increase in surface roughness is observed in benzo(b)thiophene-2-carboxaldehyde (Fig. 3d) following the addition of creatinine (Fig. 3c), with the Rq rising from 1.158 nm (pure BTCA) to 2.556 nm (BTCA + creatinine). This increase suggests significant surface reorganization, potentially resulting from physical interactions such as hydrogen bonding, π–π stacking, or molecular aggregation between BTCA and creatinine. Similar phenomena have been reported in sensing systems where hydrogen bonding and π–π stacking lead to aggregation and morphological change of the sensing film^18,19^.

**Fig. 4:**
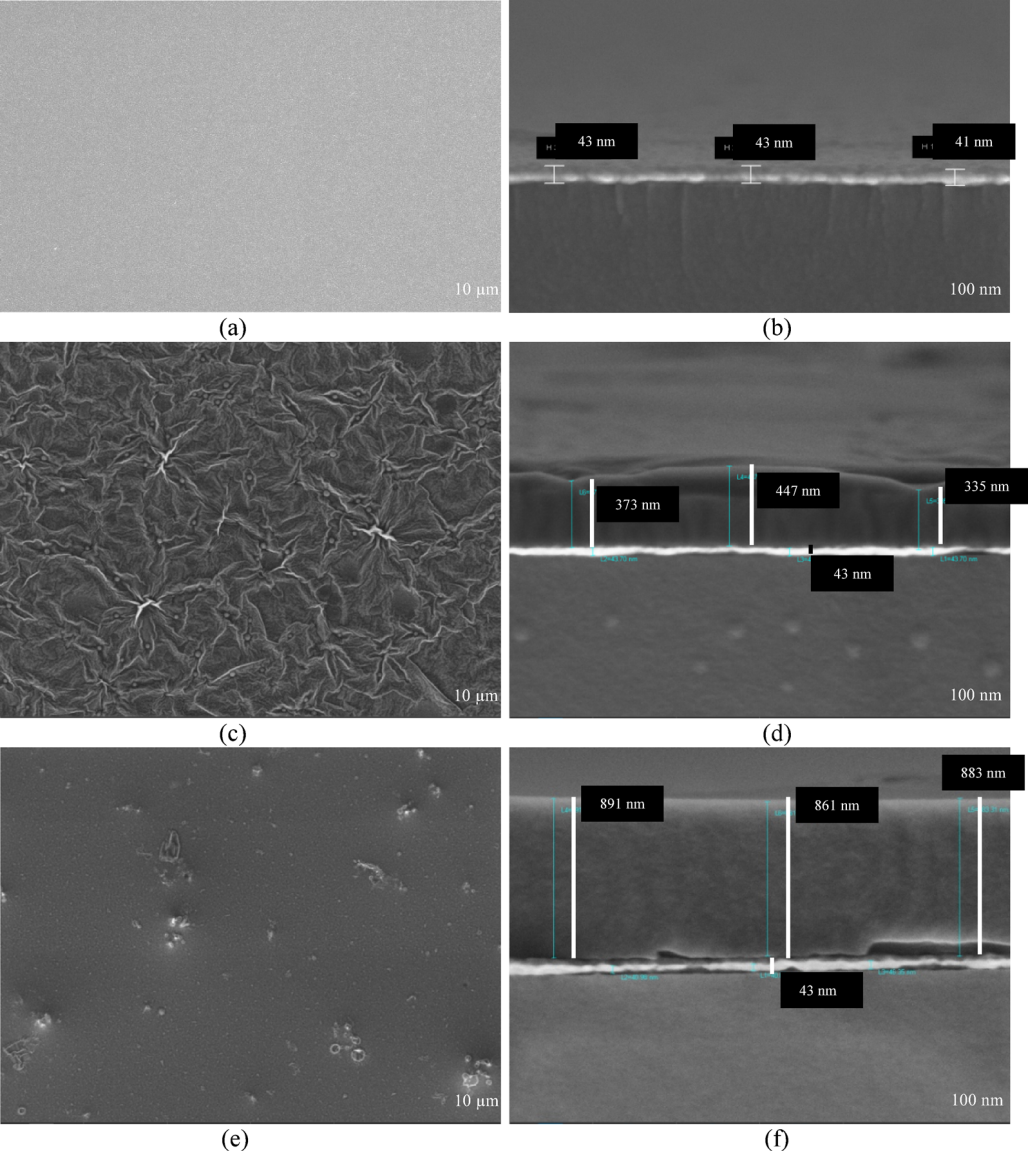
FESEM surface morphology images of **(a)** bare gold film, **(c)** THT film, and **(e)** BTCA film, together with corresponding cross-sectional images of **(b)** gold film, **(d)** THT, and **(f)** BTCA layers on the gold substrate.

**Fig. 5:**
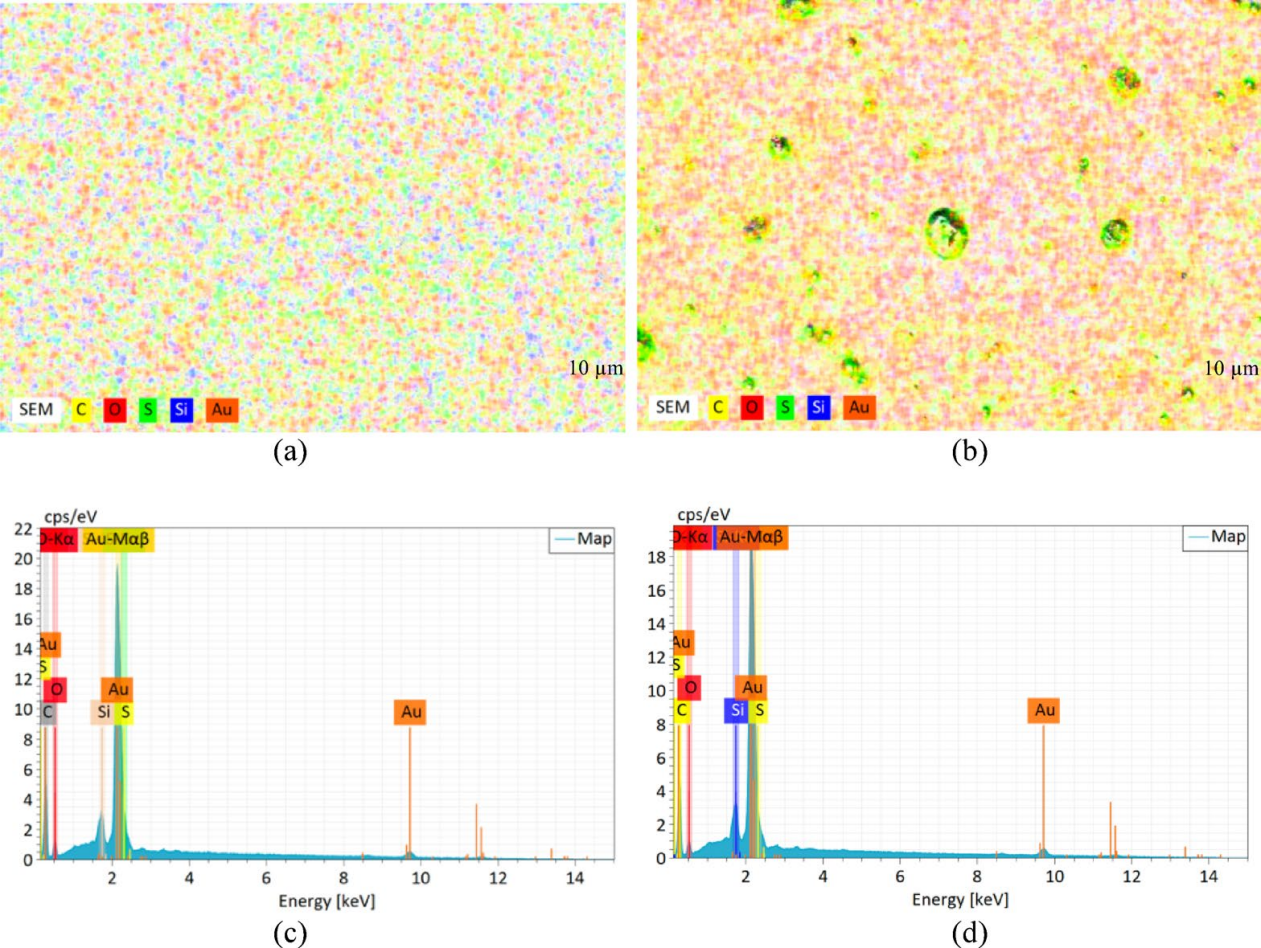
Elemental mapping and spectra images of gold films modified with (**a**, **c**) THT and (**b**, **d**) BTCA. The color distributions correspond to carbon (yellow), oxygen (red), sulfur (green), silicon (blue), and gold (orange).

Next, FESEM images show the THT and BTCA sensing layer and cross-sections, which were coated and thermally annealed on gold substrates, as well as the bare gold film. Figure 4(a) shows a smooth and uniform topography, indicating that the sputtering process resulted in a continuous and defect-free surface. This morphological homogeneity is advantageous as it provides a stable and reproducible platform for subsequent molecular coating. The cross-sectional view (Fig. 4(b)) further confirms that the gold layer is uniformly deposited with a consistent thickness of 43.54 nm. Figure 4(c) reveals that the THT film adopts a wrinkled, stacked morphology, possibly resulting from film contraction and molecular rearrangement during the thermal annealing or solvent evaporation process. This wrinkled structure suggests a high degree of molecular packing, which may enhance film adhesion to the gold substrate, like thermally processed polymer films^20^. Figure 4(d) shows the presence of an additional overlayer on top of the gold substrate, with an estimated thickness ranging from 330 to 450 nm. The THT film exhibits variations in thickness, with the thicker, ridged regions reaching ~ 450 nm, while the thinner troughs measure ~ 330 nm. Additionally, elemental analysis in Table 1 confirms the successful deposition of THT on the gold film, with the composition containing 15.1% carbon, 6.5% sulfur, and 3.8% oxygen by weight, as shown in Fig. 5(d). The detectable sulfur signal confirms the attachment of thiophene groups, consistent with the observations in the mapping image in Fig. 5(a).

However, Fig. 4e shows that BTCA forms a relatively smoother and more uniform surface, characterized by minimized granular features and wrinkled domains. Such a morphology may result from the planar and rigid nature of the benzo[b]thiophene moiety and the polar aldehyde functional group, which could facilitate more ordered molecular packing. The cross-sectional FESEM image of the BTCA film (Fig. 4f) reveals a relatively smoother and more compact overlayer compared to the wrinkled THT coating. The BTCA layer exhibits a consistent thickness of ~ 880 nm, with only minor fluctuations across its surface. This more uniform profile indicates that BTCA forms a densely packed and coherent film on the gold substrate, minimizing irregularities in film thickness^21^. Consistent with observations in conjugated organic semiconductors, where morphology and polymorphism impact interfacial transport and signal transduction, we investigate the relationship between surface organization and sensing behavior^22^. Comparable morphologies have been observed in donor–acceptor thiophene-based systems, such as BTT used in organic solar cells, where ordered domains contribute to enhanced interfacial interactions and charge transfer^20,23^. While BTCA’s morphology here is not directly linked to charge transfer, the ordered surface may favor uniform adsorption and stability of the sensing layer. Similarly, elemental mapping (Fig. 5b) confirms the deposition of BTCA on gold, showing 21.8% carbon, 4.3% sulfur, and 2.9% oxygen by weight as shown in Fig. 5d. Elemental mapping also reveals granular domains across the surface, which is consistent with the morphology seen in the surface morphology FESEM image. The combined AFM, FESEM, and EDX analyses provide supporting evidence that both THT and BTCA undergo morphological changes when exposed to creatinine. The overall increase in surface roughness and distinct topographical features after creatinine binding reflects underlying molecular interactions that affect film uniformity and packing density. These morphological changes are closely related to SPR performance, as they influence analyte diffusion, surface coverage, and ultimately the reproducibility and sensitivity of the optical signal Table 1.

**Table 1:** EDX analysis of THT and BTCA.

Condition	THT (Wt%)	BTCA (Wt%)
C	15.1	21.8
S	6.5	4.3
O	3.8	2.9

### Sensing study

**Fig. 6:**
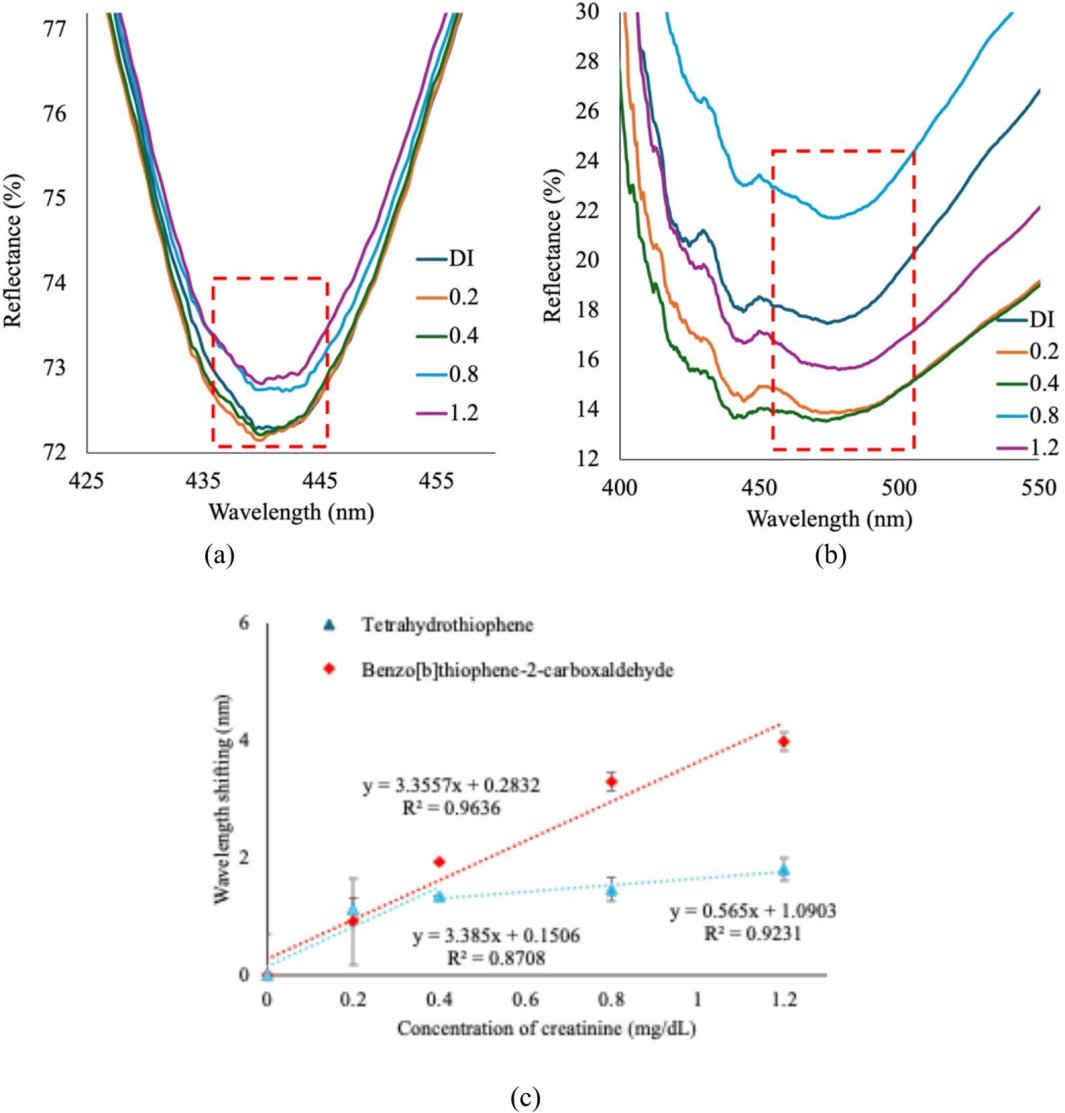
SPR spectrum (**a**) THT and (**b**) BTCA with different creatinine concentrations and (**c**) standard curve of SPR for creatinine within the 0–1.2.2 mg/dL range.

Gold FTIR spectra in Fig. 6a&b show the reflectance spectra of the SPR sensor coated with THT and BTCA films, respectively, upon exposure to increasing concentrations of creatinine ranging from 0 to 1.2 mg/dL. In both cases, a concentration-dependent shift in the resonance dip is observed, reflecting changes in the local refractive index at the sensing interface due to analyte binding. For the THT-based sensor (Fig. 6a), the resonance dip shifts from 435 to 445 nm, indicating a red shift associated with enhanced surface interaction. A similar redshift trend is observed for BTCA (Fig. 6b), with resonance dips shifting within the 460–490 nm range, suggesting stronger or more extensive binding events. Interestingly, while BTCA shows a generally progressive redshift with increasing creatinine concentration, a slight blueshift occurs at lower concentrations (0.2–0.4 mg/dL). This deviation from linear behavior may result from early-stage adsorption effects, where weak, initial interactions perturb the hydration layer or displace loosely bound surface molecules, temporarily lowering the local refractive index^24^. Such effects are often attributed to the preferential binding of creatinine to specific functional groups, such as aldehydes or thiophene sulfur, which may induce transient conformational changes before equilibrium binding is established^25^. This interpretation is supported by FTIR evidence of hydration-sensitive vibrational shifts and AFM data indicating surface reorganization upon exposure to creatinine. These findings support the role of surface chemistry in modulating the optical response, particularly in low-concentration regimes where molecular orientation, hydration effects, and functional group specificity critically influence sensor behavior. Furthermore, although the BTCA layer formed a smoother and denser surface with minimized granular features, its aldehyde and aromatic functionalities enable multiple interaction pathways, including hydrogen bonding, dipole–dipole interactions, and π–π stacking. At lower concentrations (0.2–0.8 mg/dL), these interactions occur in a competitive or partially reversible manner, resulting in irregular fluctuations in the reflectance baseline. A clearer red shift was observed at 1.2 mg/dL, consistent with stronger and more stable binding. This behavior suggests that BTCA undergoes multi-site binding and charge-transfer interactions before reaching a stable adsorption regime, which explains the observed irregularity.

The data from these reflectance shifts were used to generate the calibration plot shown in Fig. 6c, which relates wavelength shift to creatinine concentration. Error bars represent the standard deviation from three independent measurements for each concentration^21^. This curve facilitates the assessment of sensitivity, limit of detection, and limit of quantification for the SPR sensor developed with sensing film. The curve of the THT film shows a two-phase linear response, whereas the BTCA film shows only one linear response. For the THT film, at low concentrations (0–0.4 mg/dL), the response shows a steep increase with a linear fit of

$$\:\text{y}=3.385\text{x}+0.1506$$

and a correlation coefficient of R^2^ = 0.8708, indicating high sensitivity in the trace detection range. Beyond 0.4 mg/dL, the response transitions into a more gradual slope, represented by the linear equation of

$$\:\text{y}=0.565\text{x}+1.0903$$

with R^2^ = 0.9231, suggesting reduced sensitivity likely due to surface saturation effects on the sensing film, indicating a saturation effect, where further creatinine adsorption results in smaller changes in the refractive index. The saturation trend at elevated concentrations is linked to surface binding constraints, as the adsorption sites on the sensing film get filled, diminishing subsequent plasmonic shifts. The sensitivity is the change in resonance wavelength per unit variation in creatinine concentration. It is calculated by taking the derivative of Eqs. (2) and (3), yielding values of approximately 3.385 nm/mgdL^−1^ and 0.565 nm/mgdL^−1^, respectively, at near-zero concentrations. To evaluate sensor performance, the limit of detection (LOD) and limit of quantification (LOQ) were calculated separately for each concentration region based on the standard deviation of replicate measurements. For the lower concentration range (0–0.4 mg/dL), the sensor achieved LOD of 0.57 mg/dL and LOQ of 1.72 mg/dL. In the higher concentration range (above 0.4 mg/dL), the corresponding limit of detection and quantification values were 1.4 mg/dL and 4.32 mg/dL, respectively. Thus, the sensor’s most analytically precise performance occurs at higher concentrations, whereas its most sensitive response (larger shifts per unit concentration) occurs at lower concentrations.

A marked linear increase in wavelength shifting with a high slope and a strong correlation coefficient (R² = 0.9636) demonstrates a sensitive response of the BTCA film. The sensitivity of the sensor is approximately 3.3557 nm/mgdL^−1^ at near-zero concentration.

$$\:\text{y}=3.3557+0.2832$$

Additionally, the LOD and LOQ of the present sensor are determined to be 0.56 and 1.71 mg/dL, respectively. As shown in Table 2, the SPR sensors functionalized with thiophene derivatives exhibit competitive performance compared to existing material-based and standard detection platforms, especially in terms of detection and quantification limits. Among the tested configurations, the BTCA-coated SPR film achieved the lowest limit of detection (0.56 mg/dL) and a broad linear detection range (0–1.2 mg/dL), outperforming both the THT-based sensor and several previously reported electrochemical and optical approaches. This enhanced sensitivity and range reflect the more effective molecular recognition facilitated by BTCA. The SPR sensors incorporating BTCA and THT interfaces achieved low detection limits (0.56 and 0.57 mg/dL, respectively) with detection ranges appropriate for clinical analysis. These results highlight their potential as reliable, label-free alternatives with the performance of conventional sensing approaches. Equally, Table 2 highlights that BTCA with SPR-gold thin film has lower LOD/LOQ and a larger range than THT. The consequence of this complex binding profile is a high signal-to-noise ratio at low concentrations, which elevates the LOQ above our experimental range. Since the binding is not uniform and leads to non-linear interactions, the statistical requirements for robust quantification are not met. This outcome highlights the challenges of conducting high-precision kinetic analysis on complex systems. Despite the high LOQ, the reproducibility of the observed binding trends provides valuable qualitative insight^26,27^. Our data confirm that an interaction occurs between creatinine and the BTCA and THT film. The observed irregularities serve as a qualitative marker of the complex, multi-site nature of this binding, and the results provide a comparative basis for future studies.

The differences in SPR response between the two SPR configurations can be attributed to chemical interactions with the sensing material, creatinine. As shown in Fig. 1, additional functional groups of BTCA, including the aldehyde group (–CHO) and the aromatic system, which is a benzene ring attached to a thiophene ring, engage in more potential hydrogen bonding and dipole-dipole interactions involving electron-rich groups of BTCA and the amine or carbonyl groups in creatinine^28^. This aligns with the FTIR results, which show a significant reduction in the new peaks for aromatic C-H stretching at 3065 cm⁻¹ and C = O stretching of the aldehyde (1701 cm^−1^) in BTCA after the presence of creatinine. Conversely, THT’s saturated thioether ring limits interactions to weaker C–S/C–H perturbations, as reflected in its lower R² (0.807) and segmented linear response. BTCA’s planar structure may also promote uniform surface adsorption, consistent with its smoother morphology (Fig. 4b), while THT’s flexible ring could lead to disordered stacking. However, THT’s wrinkled morphology might offer advantages in high-surface-area applications. From a materials design perspective, BTCA provides performance for applications that require precision, linearity, and reproducibility, while THT may be advantageous in scenarios where trace-level detection is a priority.

**Table 2:** Comparison of sensor performance for creatinine detection using a materials-based and a standard method approach.

Sensing technique	Sensing materials	LOD (mg/dL)	LOQ (mg/dL)	Range (mg/dL)	Reference
Electrochemical	Reaction with 2-nitrobenzaldehyde	5.65	-	11–282	^29^
Electrochemical	Copper nanoparticle electrode	0.95	-	3.16–33.9	^30^
Colorimetry	Jaffe test on microfluid	1.4	2.0	2–32	^31^
UV/Vis spectrophotometer	Nano drop Jaffe test	5.843	-	60–180	^32^
LSPR	Gold nanorods/CTAB	0.645	0.712	0.2–1.2	^33^
SPR	Benzo[b]thiophene-2-carboxaldehyde	0.56	1.71	0–1.2.2	This work
SPR	Tetrahydrothiophene	0.57	1.72	0–0.4	This work

## Conclusion

This study demonstrates that the molecular structure of thiophene-based sensing materials plays a critical role in governing interfacial interactions and plasmonic sensing behavior in label-free creatinine detection. BTCA, with its conjugated aromatic backbone and polar aldehyde group, promotes stronger hydrogen bonding and π–π interactions with creatinine, leading to a uniform sensing interface and a highly linear, single-phase SPR response (R² = 0.9636) across the 0–1.2 mg/dL range. In contrast, THT’s saturated and less polar structure results in weaker molecular interactions, a segmented response profile (R² = 0.807 and 0.9231), and less uniform surface morphology. These observations are supported by FTIR and AFM analyses, which reveal structure-dependent changes in vibrational modes and topography upon creatinine binding. Overall, this work highlights the importance of molecular conjugation and functional group polarity in enhancing analyte recognition and signal transduction at the sensor interface. This establishes a quantitatively supported framework for designing structurally tunable and reproducible SPR sensors. While this work primarily focuses on the mechanistic level, the reproducible response, linear correlation, and spectroscopic validation indicate that the results represent a proof-of-concept foundation, rather than a preliminary trial. However, these findings should be interpreted with several constraints: measurements were performed in near-neutral aqueous media at ~ 25 °C, and molecular coverage/orientation were not quantified; FTIR was used comparatively and does not provide bond strengths; regeneration, storage stability, selectivity against common interferents, and explicit pH/temperature effects were not examined. Furthermore, the relatively high LOQ exceeding the tested range reflects the complexity of multi-site interactions rather than a limitation in detection capability. Future studies will integrate angle-resolved SPR, QCM-D, and DFT modeling to probe adsorption kinetics, employ covalent anchoring and molecular imprinting to improve selectivity, and validate sensor reproducibility in biological matrices under controlled pH and temperature conditions. Collectively, these efforts aim to advance thiophene-based materials from molecular understanding toward clinically reliable, label-free plasmonic diagnostics.

## Data Availability

The datasets used and/or analyzed during the current study are available from the corresponding author upon reasonable request.
